# Effect of the Temperature on Interfacial Properties of CO_2_/H_2_ Mixtures Contacting with Brine and Hydrophilic Silica by Molecular Dynamics Simulations

**DOI:** 10.1021/acs.energyfuels.3c03164

**Published:** 2023-11-08

**Authors:** Cheng Chen, Jun Xia, Hamid Bahai

**Affiliations:** Department of Mechanical and Aerospace Engineering, Brunel University London, Uxbridge UB8 3PH, United Kingdom

## Abstract

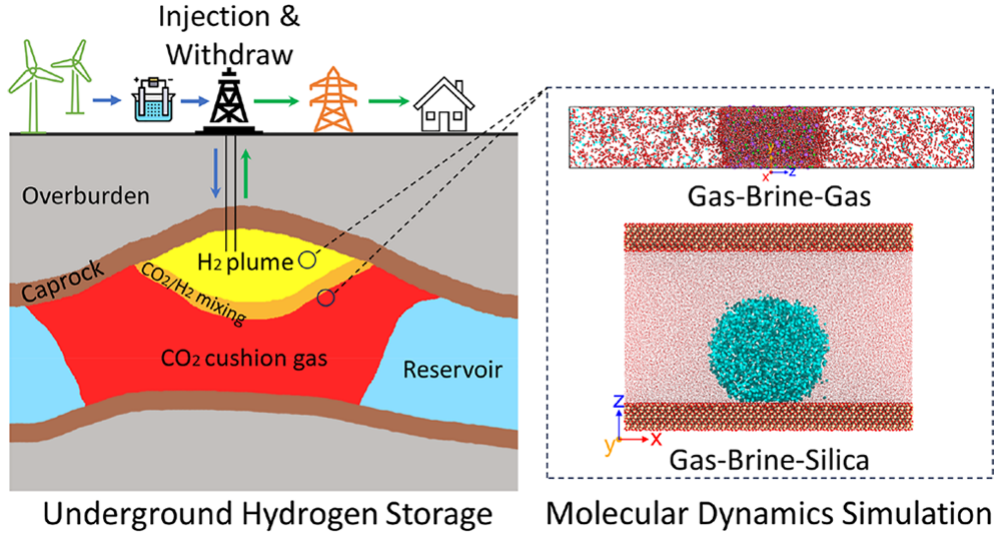

Underground H_2_ storage (UHS) is a promising technology to achieve large-scale, long-term H_2_ storage. Using CO_2_ as a cushion gas to maintain the pressure of the reservoir and withdraw stored H_2_ in the saline aquifer simultaneously enables the implementation of UHS and underground CO_2_ storage (UCS). The difference in the molecular properties of CO_2_ and H_2_ leads to distinct interfacial behavior when in contact with the brine and rock, thereby affecting the flow patterns and trapping mechanisms of gases in geological formations. Accurate prediction of the interfacial properties of CO_2_, H_2_, and the mixtures when interacting with brine and rock is crucial to minimizing the uncertainties in UHS and UCS projects. In this study, molecular dynamics (MD) simulations are performed to predict the interfacial tension, surface excess, bubble evolution, and contact angle of CO_2_, H_2_, and the mixtures at 10 MPa and 300–400 K. The MD results show that the interaction of CO_2_ with H_2_O and hydrophilic silica is considerably stronger than that of H_2_. The interfacial tension reduces linearly with the temperature in H_2_-dominated mixture systems, and the surface adsorption of H_2_ can diminish in a CO_2_-dominated system or at high-temperature conditions. The hydrophilic silica is more CO_2_-wet than H_2_-wet, and the attached CO_2_ bubble is more easily disconnected. Ions and the temperature play different roles in the contact angle.

## 1 Background

CO_2_ capture, utilization, and storage (CCUS) plays a key role in achieving net zero. According to the International Energy Agency (IEA) assessment of the net-zero trajectories, the demand for CO_2_ storage is projected to escalate significantly from approximately 40 Mt/year to more than 5000 Mt/year by 2050.^1^ The underground CO_2_ storage (UCS) is the vital cornerstone of the CCUS value chain, in which captured CO_2_ is compressed, transported, and injected into the deep undersurface reservoir and finally trapped by the geological porous formations. According to the IEA estimation, the global UCS capacity can be 8000–55 000 Gt.^2^ More recently, injecting H_2_ into the reservoirs and achieving the underground H_2_ storage (UHS) is proposed to meet the demand of large-scale and long-term (GWh/TWh and weeks–seasons) storage. Some pilot projects show the feasibility of UHS. For example, a mixture of 3–4% CO_2_ and 95% H_2_ has been stored in a salt cavern in Teesside, U.K., at the depth of 400 m.^3^ During the UHS process, CO_2_ can be used as cushion gas to maintain the pressure and withdraw stored H_2_. This indicates that CO_2_ working as a cushion gas in UHS is a new CO_2_ utilization scenario, which can facilitate the deployment of UCS and UHS simultaneously.

The potential site options for underground gas storage (UGS) mainly include depleted gas/oil reservoirs, coal seams, deep saline aquifers, and salt caverns, which differ by their location, quantity, formation component, seal property, and storage capacity. Although a salt cavern is relatively impermeable and has a high level of containment integrity, it has limited quantity and smaller storage capacity.^3^ Depleted gas/oil reservoirs and saline aquifers are porous media containing considerable amounts of nanopores,^4^ and deep saline aquifers exhibit a potential capacity of 2000–13 000 Gt.^2^ The injection of a large volume of CO_2_ into deep saline aquifers will displace the resident fluids and induce the multiphase fluid flow, followed by the solute transport and chemical reactions between fluids and formation minerals. To be specific, the injected gases would flow upward as a result of the buoyant effect and the density difference until reaching the impermeable layer of tight caprock, which is known as the structural trapping. As CO_2_ continues to migrate within the aquifer, a fraction of it can remain as isolated or residual bubbles or droplets in the pore spaces. The CO_2_ plume is split into numerous micrometer-scale bubbles or droplets, which are immobilized by the capillary forces within the pore network of the rock, which is referred to as the residual trapping.^5^ At the interface between CO_2_ and brine, a portion of CO_2_ can dissolve into the brine and generate mild carbonic acid (H_2_CO_3_). Bicarbonate can react with the minerals and transform into solid carbonate minerals, achieving permanent trapping. These four different trapping mechanisms (structural trapping, residual trapping, dissolution trapping, and mineral trapping) account differently for storage capacity and safety. Unlike UCS, the dissolution trapping and geochemical reactions, such as methanogenesis in UHS, can lead to the significant loss of H_2_ which are absolutely unfavorable.^6^

The intermolecular interaction of the gas–liquid–rock system determines the capillary entry pressure (*P*_ce_) required for gas penetration in the pores. *P*_ce_, in turn, significantly affects the structural trapping capacity of the caprocks and the efficiency of residual trapping in deep saline aquifers.^5,7,8^*P*_ce_ can be calculated by the Young–Laplace equation
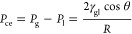
1where *P*_g_ is the pressure of the gas phase, *P*_l_ is the pressure of the liquid phase, γ is the interfacial tension (IFT) of gas and liquid, θ is the contact angle (CA), and *R* is the pore through radius.

The maximum column height, *h* (i.e., volume), of gas immobilized beneath the caprock^7,9^ is expressed as
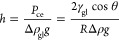
2where Δρ is the gas–liquid density difference and *g* is the gravitational constant.

Clearly, it is crucial to accurately predict the basic properties, such as the density, IFT, and CA, at reservoir conditions for reliable evaluations of the UCS and UHS projects. In general, the conditions of the saline aquifer vary with the depth following roughly that *T* (°C) = 15 + 33*d* and *P* (atm) = 1 + 100*d*, where *d* is the depth in kilometers.^10^ The typical conditions of UCS and UHS are in the range of 300–400 K and 5–25 MPa;^5,9,11^ thus, CO_2_ (*T*_c_ = 304.13 K and *P*_c_ = 7.38 MPa) is in liquid or supercritical conditions, while H_2_ is more likely in its supercritical conditions (*T*_c_ = 33.15 K and *P*_c_ = 1.30 MPa). Captured CO_2_ from the steam methane reforming process or from a steel mill can always have impurities, such as H_2_S, CH_4_, and H_2_. The impurities in CO_2_ can affect its solubility, injectivity, and storage capacity.^12−14^ It was found that the difference among H_2_, CO_2_, and H_2_O regarding the density, viscosity, IFT, and wettability can lead to the complicated flow patterns and displacement process, such as the viscous/capillary fingering structures.^3,15,16^ However, to the best of our knowledge, the interfacial properties of CO_2_/H_2_ mixtures in contact with brine and rock are extremely lacking.

A substantial number of experiments and modeling studies, mainly the molecular dynamics (MD) simulation, have been performed in context of UCS. More recently, a few unsystematic works were also performed for UHS. For example, vapor–liquid equilibrium (VLE) MD simulations have been performed for the CO_2_–brine/oil systems^17,18^ and H_2_–brine system^19^ with pressure up to 100 MPa, which accurately predicted the surface excess adsorption and the IFT. Although having huge uncertainties in experiments,^20^ the consensus is reached that CA of water increases with the pressure of CO_2_ because the increased CO_2_ density can enhance the CO_2_–rock intermolecular interactions.^5^ MD simulation results of the CA using the CO_2_–H_2_O (in sessile droplet)–rock system at pressures up to 50 MPa^11,21^ also follow the trend. In the experimental measurement of the water CA in the H_2_–brine–clay system using the sessile droplet method, it was found that cos(θ) correlates linearly with the H_2_ density when contacting with some typical clay mineral slabs, such as montmorillonite, illite, and kaolinite.^22,23^ However, the MD simulation by Al-Yaseri et al.^24^ found that H_2_ is completely non-wetting and CA is independent of the H_2_ pressure in both H_2_–H_2_O–quartz and H_2_–H_2_O–calcite systems, and they emphasized the necessity to clean the mineral samples to avoid the surface contamination-induced uncertainties. The research on UHS is still in its infancy, and reliable interfacial properties of H_2_ contacting with brine and rock are highly needed.^9^ The effect of the temperature on CA is still an open question, and more research is highly needed to understand the mechanism.^5,22,25^

High-pressure, wide-range temperature conditions cause many difficulties in experiments particularly considering the flammability of H_2_. In this study, MD simulation is performed to investigate the effect of the temperature (300–400 K) on interfacial properties of the CO_2_/H_2_–brine system and the CA of the brine–CO_2_/H_2_–rock system using the captive bubble method at an isobaric condition of 10 MPa. The paper is organized as follows: molecular models and force fields of the components, including CO_2_, H_2_, H_2_O, NaCl, and a silica model representing the hydrophilic rock, are given in section 2.1. The system configuration and MD setup of the gas–brine two-phase system and gas–brine–rock three-phase system are given in section 2.2. The results of interfacial properties of the two-phase system are given in section 3.1. The effect of the bubble size on morphology evolution is given in section 3.2. The effect of the temperature on CA is given in section 3.3. Finally, the conclusion is given in section 4.

## 2 MD Setup

### 2.1 Molecular Model and Force Field

The intra- and intermolecular interactions are described by the force field. In this study, the transferable potentials for phase equilibria (TraPPE) force field developed by Potoff et al.^26^ was used for CO_2_ molecules, with flexible bonds and angles. The parameters of bond stretch and angle bend are taken from the work of Zhong et al.,^27^ which have been validated against experimental data on transport properties over a wide range of conditions. The Madrid-2019 ion model developed by Zeron et al.^28^ based on the TIP4P water model^29^ was used for brine, in which the ion charge of Na^+^ and Cl^–^ is scaled by 0.85 for better description of the infinite dilution properties. It was demonstrated that this set of force fields can be used to model the practical seawater, with excellent prediction on transport properties, structural properties, and interfacial properties.^30^ The interface force field (IFF) developed by Wang et al.^31^ and Heinz et al.^32^ are used for H_2_ molecules and Q2 silica (9.4 silanol groups/nm^2^), respectively. The IFF force field has been used widely in the CO_2_–H_2_O–silcia system to predict the contact angle and wettability.^11,21^

The force fields are compatible with each other as they use the same formula to describe the potential energy of the MD systems, expressed as
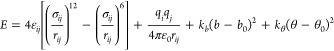
3where the first two terms are the non-bonded interactions of van der Waals (vdW) force and electrostatic force, while the last two terms are intramolecular energies of the bond stretch and angle bend, *i* and *j* are indices of atoms, ε and σ are energy and size parameters of the Lennard-Jones (LJ) potential, *q* is the charge of the atom, ε_0_ is the vacuum permittivity, *r*_*ij*_ is the distance between two atoms *i* and *j*, *k*_r_ and *k*_θ_ are the energy constants, *b* and θ are the bond length and angle of two bonds, and *b*_0_ and θ_0_ are the equilibrium values. The other intramolecular energies of dihedral and out of plane are not considered for silica.^33^ The interaction of different particle types is described by the Lorentz–Berthelot combining rules: σ_*ij*_ = (σ_*ii*_ + σ_*jj*_)/2 and ε_*ij*_ = (ε_*ii*_ε_*jj*_)^1/2^.

### 2.2 MD Setup and System Configuration

All MD simulations are performed using the package of Large-scale Atomic/Molecular Massively Parallel Simulator (LAMMPS),^34^ and the visualization is performed using the Open Visualization Tool (OVITO).^35^ The cutoff distance for vdW and electrostatic interactions is 1.4 nm. The particle–particle particle–mesh (PPPM) solver with an accuracy of 1 × 10^–5^ is used to compute the long-range electrostatic interaction.

**Figure 1:**
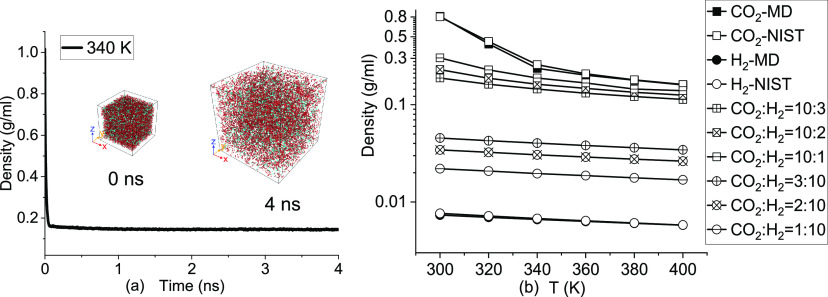
(a) Time evolution of the density and snapshots of the bulk MD system containing 10 000 CO_2_ and 3000 H_2_ molecules. (b) Effect of the temperature on the density of CO_2_, H_2_, and CO_2_/H_2_ mixtures.

The bulk MD system is built as shown in Figure 1 to validate the force field of CO_2_ and H_2_ as well as compute the density of the gas mixture. In addition to the systems of pure CO_2_ and H_2_ gases, two other binary gas mixture systems are also built. One is dominated by CO_2_, while H_2_ is added as an impurity with the mole ratio of H_2_/CO_2_ equal to 1:10, 2:10, and 3:10. The other one is dominated by H_2_, while CO_2_ is added as an impurity with the mole ratio of CO_2_/H_2_ equal to 1:10, 2:10, and 3:10. Gases are equilibrated in the *NPT* (constant pressure/temperature, where *N* is the number of molecules, *P* is the pressure, *T* is the temperature) ensemble for 4 ns with a time step of 1 fs. Periodical boundary conditions in all three directions are implemented in the bulk MD simulation.

The system at 340 K reached equilibrium after 63 ps, as shown in Figure 1a, and the density of Figure 1b is the averaged data of the last 2 ns *NPT* simulation. The density of pure CO_2_ and H_2_ from MD simulation agrees well with the National Institute of Standards and Technology (NIST) data. The effect of the temperature on the CO_2_ and CO_2_-dominated systems is much more prominent than that of H_2_ and H_2_-dominated systems.

**Figure 2:**
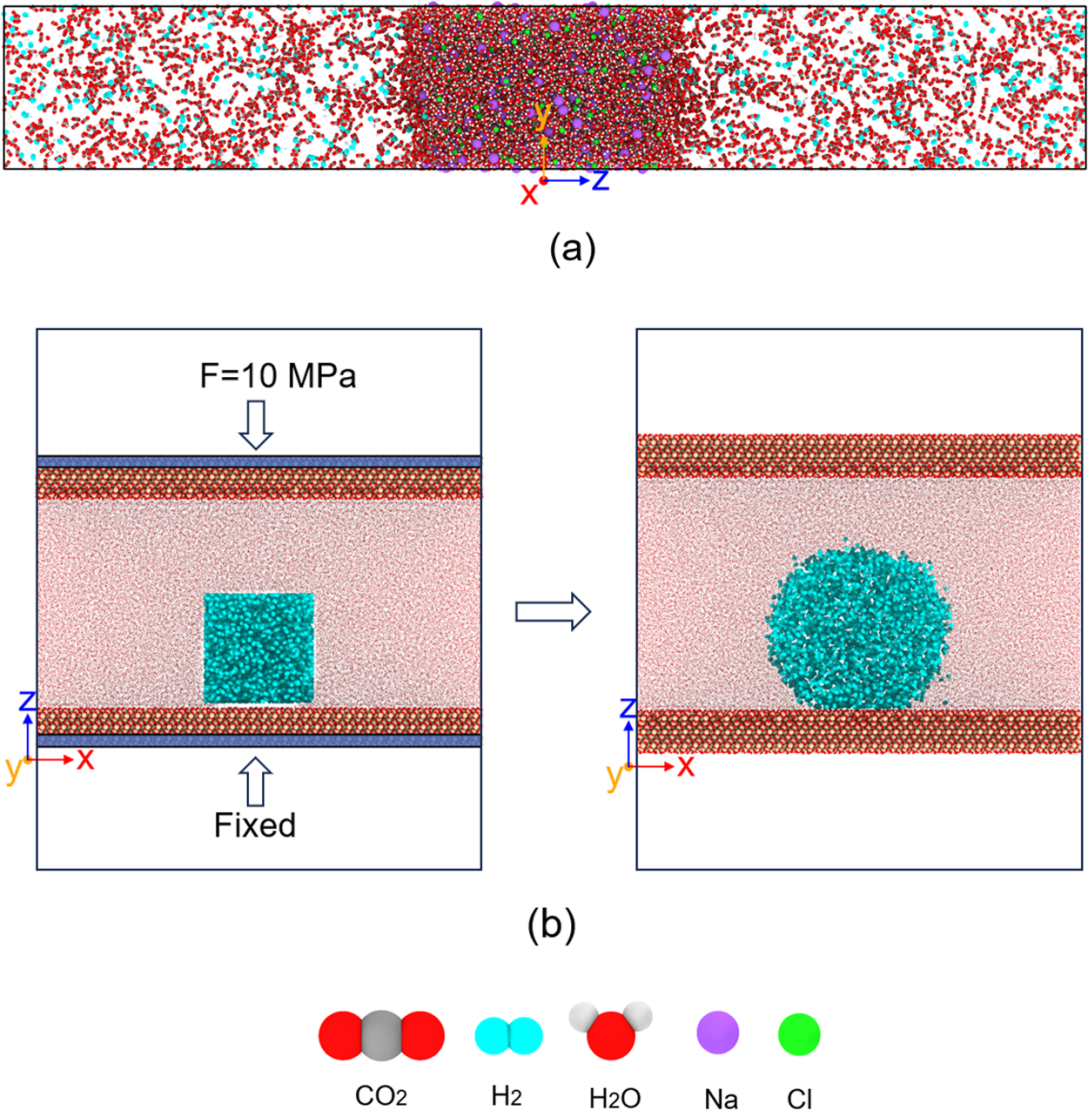
(a) Gas–brine system after equilibrium. The brine film contains 10 000 H_2_O molecules and 400 Cl^+^ and 400 Na^+^ ions. The thickness of the brine film is about 8.8 nm. The box length is (*L*_*x*_, *L*_*y*_, *L*_*z*_) = (6, 6, 40) nm. (b) Equilibrium process of the system: H_2_O–H_2_–silcia. (*L*_*x*_, *L*_*y*_) = (20.8, 10.3) nm for the Q2 slab. The number of H_2_O molecules is 60 000. The atoms in the shaded area are added via the external force. The atom vdW radius of H_2_ is scaled by a factor of 2, while the atom radius of H_2_O is scaled by 0.2 for visualization.

The gas–brine two-phase system and brine–gas–silica three-phase system are built to study the interfacial properties, as shown in Figure 2. The mole ratio of the ion with H_2_O is 4% (2.22 M in molality), which is relatively higher than seawater considering that the evaporation-induced salt precipitation would always occur during the injection. For the two-phase system as shown in Figure 2a, the periodical boundary conditions are implemented in all directions. The box length is the same in all systems, and the number of gas molecules is calculated and packed into the box according to the gas density in Figure 1 to keep the pressure at about 10 MPa.

The number of gas molecules varies in the three-phase system, as shown in Figure 2b, to study the effect of the bubble size on morphology evolution. The pressure is controlled by adding external forces on portion atoms of the top slab, while the position of the bottom slab is fixed.^36^ The periodical boundary conditions are implemented in *x* and *y* directions, while fixed non-periodical boundary conditions are used in the *z* direction with a vacuum space of 5 nm to virtually turn off the interactions between the slabs.

## 3 Results

### 3.1 Effect of the Temperature on Interfacial Properties of the Gas–Brine System

**Figure 3:**
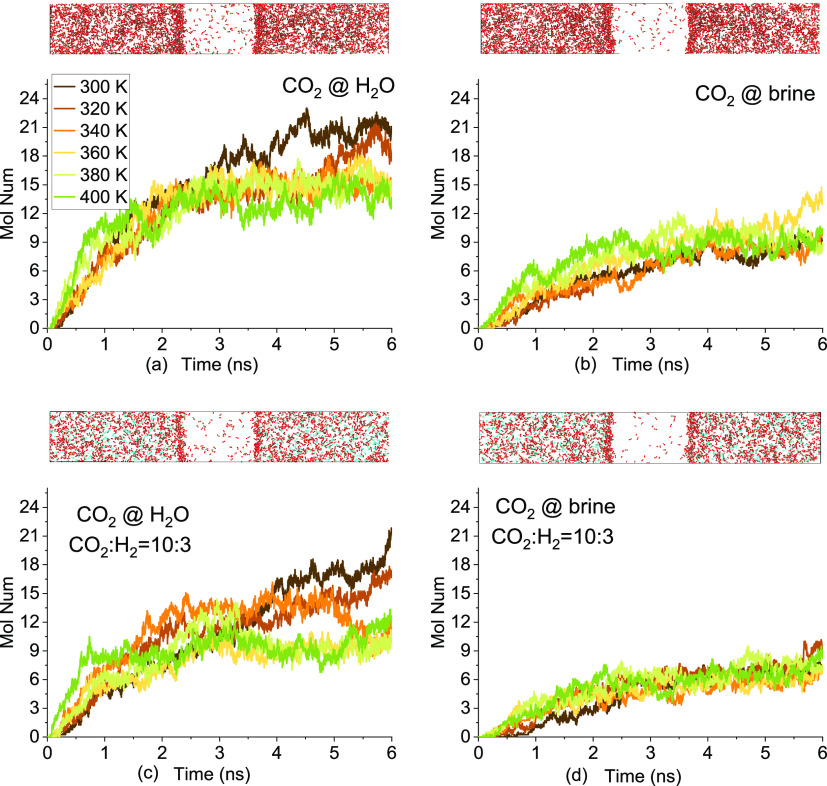
Time evolution of the CO_2_ molecule number in the region of −2.5 < *z* < 2.5 nm in different systems: (a) CO_2_ contacting with H_2_O, (b) CO_2_ contacting with brine, (c) CO_2_ and H_2_ mixtures contacting with H_2_O (gas molar ratio of CO_2_/H_2_ = 10:3), and (d) CO_2_ and H_2_ mixtures contacting with brine (gas molar ratio of CO_2_/H_2_ = 10:3). Snapshots are at 6 ns and 340 K. Brine molecules are hidden.

The time evolution of the CO_2_ molecules in the middle brine film region of −2.5 < *z* < 2.5 nm is shown in Figure 3. CO_2_ molecules accumulate at the interface and diffuse into H_2_O films after they reach saturation, as shown in Figure 3a. A higher temperature indicates higher diffusivity but lower solubility, as CO_2_ molecules reach a plateau much faster at 400 K than 300 K, with the value almost been halved. This agrees well with the work of Shiga et al.,^37^ in which the solubility of CO_2_ reduced by about 50% at 10 MPa when the temperature is increased from 300 to 400 K. H_2_ can also accumulate slightly or even be unidentifiable at the interface, and only a few H_2_ molecules diffuse into the middle region (not shown). NaCl slows the diffusion and reduces the solubility of CO_2_, as shown in Figure 3b. The effect of the temperature on CO_2_ solubility in brine is not as evident at that in H_2_O. Adding 30% H_2_ into CO_2_ does not evidently alter the diffusivity and solubility of CO_2_ in both H_2_O and brine.

**Figure 4:**
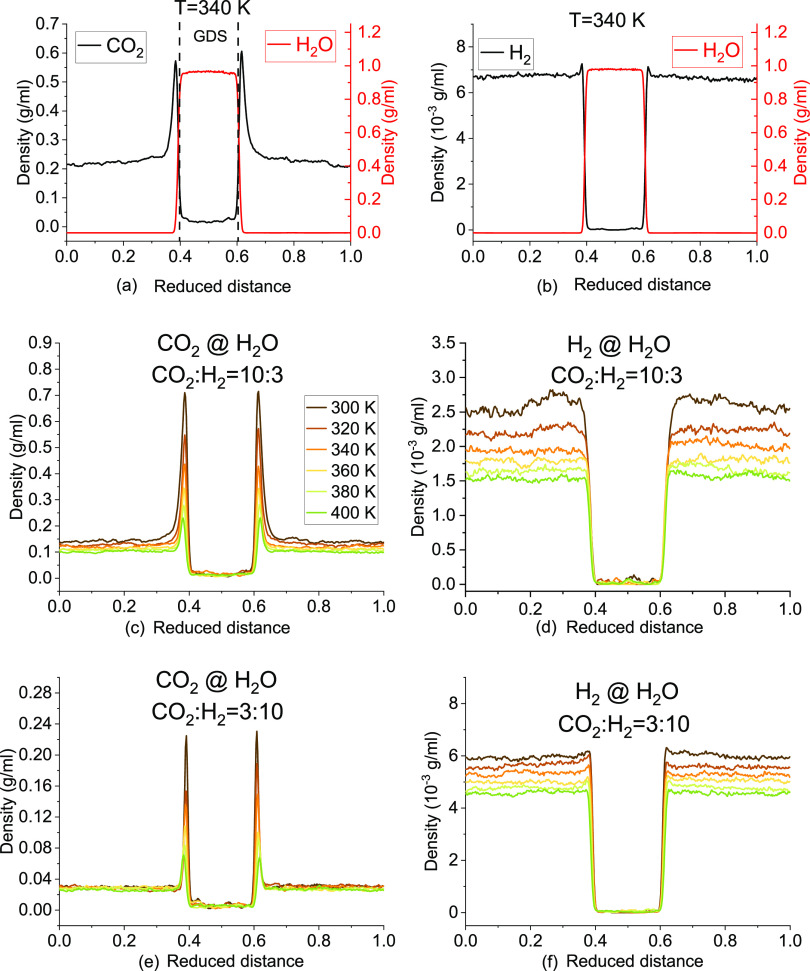
Density profile of CO_2_, H_2_, and H_2_O in different systems: (a) CO_2_–H_2_O system at 340 K, (b) H_2_–H_2_O system at 340 K, and (c–f) CO_2_/H_2_ mixtures–H_2_O system at different temperatures. The dashed lines in panels a and b are to indicate the position of the GDS.

After systems reach equilibrium, the density profile ρ_*i*_(*z*) of the gases and water is computed and demonstrated in Figure 4. The surface excess (Γ) and enrichment (*E*) are used to characterize the gas–liquid interfacial adsorption property based on the density profile. The surface excess of component *i* relative to *j* (Γ_*i*,*j*_) is used to quantify the relative adsorption tendency of *i* to *j* in the interface, expressed as^38−40^
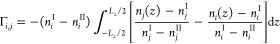
4or specifically gas *i* to water in Figure 4 can be computed by the following equation:^39^
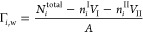
5where *n* is the number density, *N* is the total number of gas molecules, I and II denote the gas-rich bulk phase and water-rich bulk phase, distinguished by the Gibbs dividing surface (GDS), *V* is the volume of phase I or II, and *A* is the area of the interface. The GDS is used to identify the position of the interface where the surface excess of water is zero from the density profile of water;^38,39^ i.e., Γ_w,w_ = 0.

The interfacial enrichment of component *i* is defined as^40^
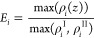
6At 340 K, the density of water increases monotonously from vapor to liquid phase, while the density of the gases exhibits a peak at the interface for both CO_2_ and H_2_ because of the surface activity.^41^ The enrichment of CO_2_ is much higher than that of H_2_ at 340 K, and CO_2_ has a thicker adsorption layer than H_2_. In the CO_2_/H_2_ mixture system, the enrichment of CO_2_ is enhanced, while that of H_2_ is reduced. In the CO_2_-dominated system, the surface adsorption of H_2_ at the interface disappears because the value of *E*_H_2__ can be less than 1. The enrichment of both CO_2_ and H_2_ also decreases with the temperature in the mixture system, and H_2_ enrichment disappears at 400 K in the system of CO_2_/H_2_ = 3:10.

**Figure 5:**
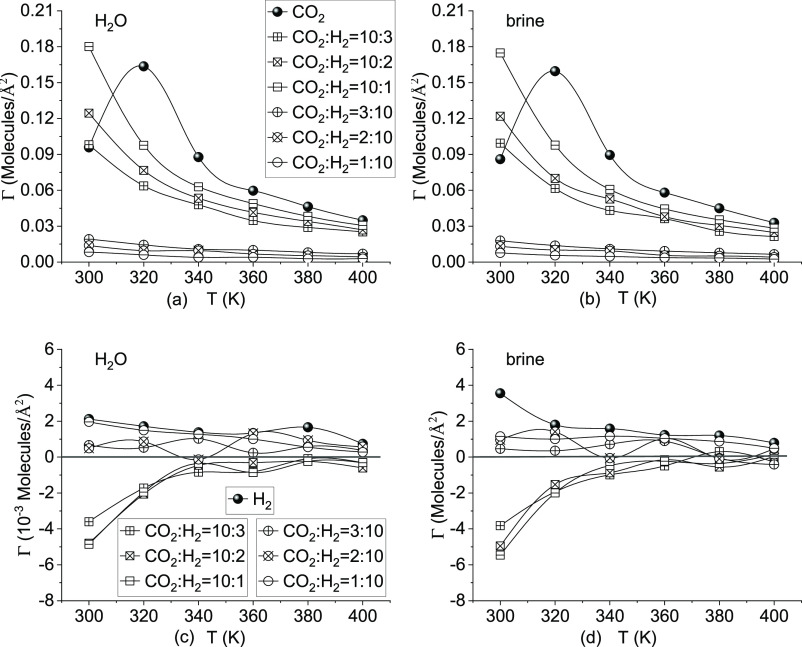
Effect of the temperature on the surface excess of gases: (a and b) surface excess of CO_2_ and (c and d) surface excess of H_2_.

The enrichment and surface excess are linked but do not contain the same information.^40,41^ The surface adsorption of CO_2_ and H_2_ in different systems is shown in Figure 5. The CO_2_ surface excess values are always positive, while the values of H_2_ can be negative. The difference between the CO_2_ and H_2_ adsorption property is because CO_2_ is quadrupole, which is capable of establishing a Debye interaction with H_2_O, while there is only a weak vdW interaction between H_2_ and H_2_O. For pure CO_2_, the surface excess increased to the peak value at 320 K and then decreased with the temperature. This agrees well with the work of Shiga et al.^37^ that the surface excess of CO_2_ at isobaric conditions always has the positive peak value appearing at the phase transition temperature when the pressure is less than 20 MPa. In a mixture system, the surface excess reduces gradually with the temperature, and the values for CO_2_-dominated systems are much higher than those of H_2_-dominated systems. The increase in the H_2_ concentration can reduce the surface excess of CO_2_. The surface excess of H_2_ reduces with the temperature at pure H_2_ and H_2_-dominated mixture systems, with the values about 10^–2^ smaller than those of CO_2_. In the CO_2_-dominated system, the surface excess of H_2_ is negative, increases gradually with the temperature, and reaches zero at about 340–360 K. The NaCl ions have limited effects on the surface excess.

**Figure 6:**
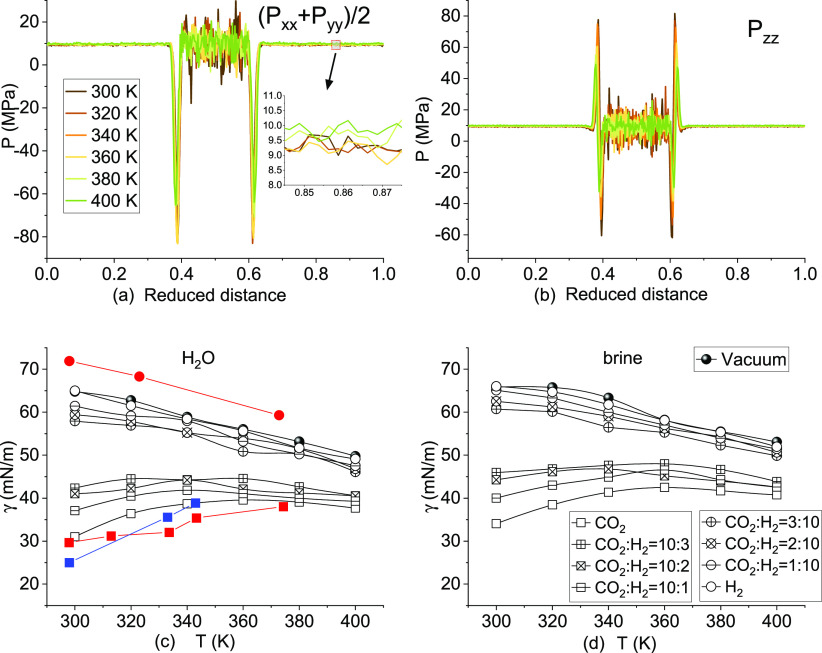
(a and b) Profile of the tangential and normal pressure components of the system CO_2_/H_2_ = 10:3 at H_2_O. (c and d) Effect of the temperature on the IFT of different systems. The red solid circles are experimental data of the H_2_–H_2_O system adapted with permission from ref (47). Copyright 2018 Elsevier. The red solid squares are experimental data of the CO_2_–H_2_O system adapted with permission from ref (48). Copyright 2010 American Chemical Society. The blue solid squares are experimental data of the CO_2_–H_2_O system adapted with permission from ref (25). Copyright 2015 Elsevier.

Besides the density profile, the pressure profile and IFT of the gas–brine system are calculated and shown in Figure 6. The pressure profile in panels a and b of Figure 6 is calculated by the summation of the per atom stress tensor in each bin. The method developed by Irving–Kirkwood^42^ is used to compute the IFT expressed in terms of the difference between the normal and tangential components of the pressure tensor^43^
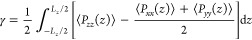
7or by
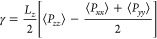
8where *P*_αα_ is the diagonal component of the pressure tensor and the pre-factor of ^1^/_2_ considers the existence of two interfaces in the simulation box.

The average pressure of the gas phase is in the range of 9.5–10 MPa, which is a bit lower than the assigned value because the surface adsorption and dissolution reduce the molecular number in the gas phase. The tangential pressure tensor exhibits strong negative values, while the normal pressure tensor has a positive maximum value and a negative minimum value at the interfaces.^44,45^ From panels c and d of Figure 6, IFTs of the brine system are higher than those of the H_2_O system, which is consistent with the previous work of Zhao et al.,^46^ who found that the incremental IFT (Δγ = γ^CO_2_–brine^ – γ^CO_2_–H_2_O^) increases linearly with the salinity. Pure H_2_ can hardly alter the IFT, while CO_2_ significantly reduces the IFT. It is because the electrostatic interactions between CO_2_ and H_2_O play an important role in virial anisotropy for the CO_2_–H_2_O system.^39^ In H_2_ and H_2_-dominated systems, the IFT decreases linearly with the temperature for both H_2_O and brine systems. Unlike the monotonous trend of the IFT with the CO_2_ pressure at isothermal conditions,^39^ the IFT increases with the temperature until it reaches the peak at about 320–360 K and then decreases with the temperature in pure CO_2_ and CO_2_-dominated systems.

### 3.2 Effect of the System Size and Gas Dissolution on Bubble Morphology

**Figure 7:**
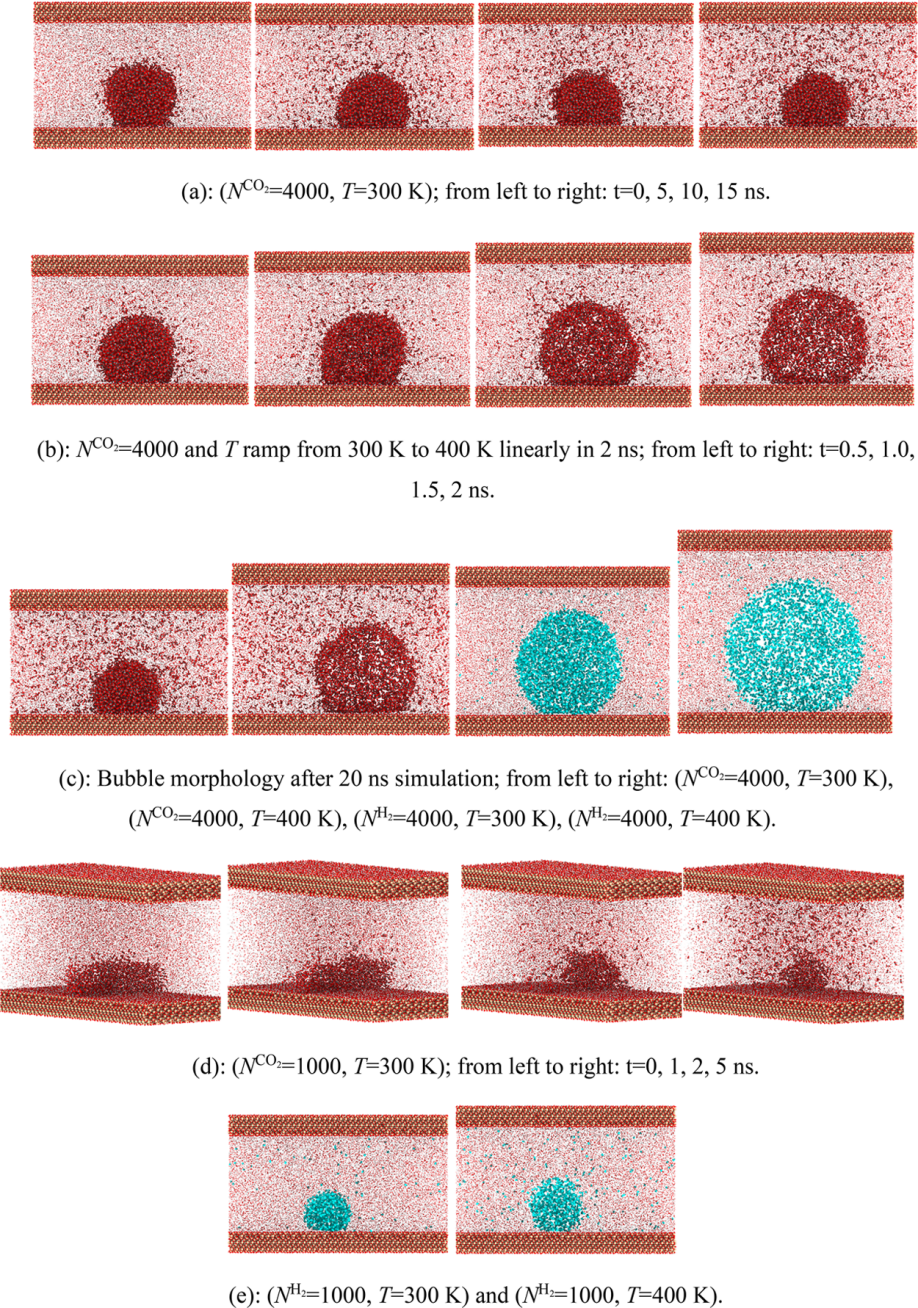
Effect of the system size and dissolution on bubble morphology at 300 and 400 K.

In MD simulation of the wettability using the sessile droplet method, it has been justified that the CA is independent of the size of the water droplet in cylindrical shape.^49^ However, the effect of the system size (indicated by the number of gas molecules, *N*) and gas dissolution on bubble morphology is still lacking. The time evolution and temperature effect on bubble morphology of gases with *N* = 4000 and 2000 are shown in Figure 7. The system is maintained at 300 K for 20 ns or increased to 400 K in 2 ns followed by a 20 ns constant temperature simulation at 400 K. As expected, the size of the gas bubble would decrease with time as a result of the dissolution but increase with the temperature as a result of the expansion. The system size of *N* = 4000 is sufficient to maintain the bubble morphology for both CO_2_ and H_2_ in 300 and 400 K even after CO_2_ dissolution completes and reaches saturation. However, the bubble would be disconnected at 300 K after 1 ns simulation in a smaller system of CO_2_ with *N* = 1000, while this does not occur for H_2_. The disconnected CO_2_ bubble changes into a semi-spherical shape and continues the dissolution.

### 3.3 Effect of the Temperature on the Contact Angle

Considering that the significant difference of the dissolution of CO_2_ and H_2_ can alter the concentration of the mixtures inside the bubble after a long time evolution, the CAs of pure CO_2_ and H_2_ bubble are summarized in Table 1. The contact angle is computed by the two-dimensional (2D) density profiles of the gas and liquid. A circular profile is fitted at the gas–liquid interface, where the isodensity is in the range of 0.2–0.4 g/mL. The contact angle is determined by , where *b* is the coordinate of the center of the fitted circle in the *z* direction, *R* is the circle radius, and *z*_0_ is the height of the contact plane.^49^

**Table 1:** CAs of CO_2_ and H_2_ at 300 and 400 K

CA (deg)		300 K	400 K
CO_2_	H_2_O	122.10	134.31
	brine	109.84	130.80
H_2_	H_2_O	155.80	156.84
	brine	146.68	151.04

The CAs of the CO_2_ and H_2_ bubbles are all greater than 100° in all conditions because the Q2 silica is strongly water-wet compared to gas-wet owing to the hydrogen bonding between water and the hydroxyl groups on the Q2 surface. The CAs of CO_2_ are smaller than those of H_2_ in the same conditions, which indicates that Q2 is more CO_2_-wet than H_2_-wet. NaCl ions can reduce the CAs because the ions can form the electrical double layer, which reduces the polarity of the silica surface, and this effect is more pronounced at a low temperature. CAs of CO_2_ increase with the temperature for both H_2_O and brine systems. This agrees with the work of Chen et al.,^50^ using a sessile droplet surrounded by CO_2_, where the CA of H_2_O at 318 K and 9.5 MPa is 33°, which is reduced to 26.4° at 383 K and 26.4 MPa. For H_2_, the effect of the temperature on CAs is not as pronounced as that of CO_2_.

## 4 Conclusion

The MD simulation is performed to predict the interfacial properties of CO_2_/H_2_ mixtures contacting with the brine film at operation conditions of 10 MPa and the temperature ranging from 300 to 400 K. The morphology of gas bubbles in the gas–brine–rock system is investigated. The CAs of the CO_2_ and H_2_ bubbles are calculated. The conclusions are drawn as follows:

The dissolution of CO_2_ in water and brine is much higher than that of H_2_. NaCl ions reduce the diffusivity and solubility of CO_2_ in the brine film. The temperature increases the diffusivity of CO_2_ but reduces its solubility. CO_2_ has a much stronger affinity than H_2_ with H_2_O at the interface, and the surface adsorption of H_2_ is not as prominent as that of CO_2_. The surface excess of H_2_ can be negative in the CO_2_-dominated mixture system. The interaction between H_2_ and H_2_O is too weak to alter the IFT under all conditions. The IFT reduces with the temperature monotonously at the H_2_-dominated mixture system, while there would be peak values at about 320–360 K in the CO_2_ and CO_2_-dominated systems. NaCl ions can increase the IFT in all systems.

To use the bubble morphology for CA calculation, the initial size of the CO_2_ bubble should be relatively larger to avoid the dissolution-induced disconnection. H_2_ is much less wet than CO_2_ on hydrophilic silica. NaCl ions reduce the CA of gas bubbles, especially at a low temperature. Unlike the CO_2_ bubble, effect of the temperature on the CA of the H_2_ bubble is not prominent.

## Data Availability

The data underpinning this publication can be accessed from the data repository of Brunel University London, Brunelfigshare, here under a CCBY license: https://figshare.com/articles/figure/Energy_and_Fuel/24282280.
